# Influence of Gender on Radiosensitivity during Radiochemotherapy of Advanced Rectal Cancer

**DOI:** 10.3390/cancers14010148

**Published:** 2021-12-29

**Authors:** Barbara Schuster, Markus Hecht, Manfred Schmidt, Marlen Haderlein, Tina Jost, Maike Büttner-Herold, Klaus Weber, Axel Denz, Robert Grützmann, Arndt Hartmann, Hans Geinitz, Rainer Fietkau, Luitpold V. Distel

**Affiliations:** 1 Department of Radiation Oncology, Universitätsklinikum Erlangen, Friedrich-Alexander-Universität Erlangen-Nürnberg, 91054 Erlangen, Germany; schuster-barbara@gmx.net (B.S.); Markus.Hecht@uk-erlangen.de (M.H.); Manfred.Schmidt@uk-erlangen.de (M.S.); Marlen.Haderlein@uk-erlangen.de (M.H.); Tina.Jost@uk-erlangen.de (T.J.); Rainer.Fietkau@uk-erlangen.de (R.F.); 2 Comprehensive Cancer Center Erlangen-EMN (CCC ER-EMN), Universitätsklinikum Erlangen, Friedrich-Alexander-Universität Erlangen-Nürnberg, 91054 Erlangen, Germany; Maike.Buettner-Herold@uk-erlangen.de (M.B.-H.); Klaus.Weber@uk-erlangen.de (K.W.); Axel.Denz@uk-erlangen.de (A.D.); Robert.Gruetzmann@uk-erlangen.de (R.G.); Arndt.Hartmann@uk-erlangen.de (A.H.); 3 Department of Nephropathology, Institute of Pathology, Universitätsklinikum Erlangen, Friedrich-Alexander-University Erlangen-Nuremberg (FAU), 91054 Erlangen, Germany; 4 Department of General and Visceral Surgery, Friedrich Alexander University, Krankenhausstraße 12, 91054 Erlangen, Germany; 5 Institute of Pathology, Universitätsklinikum Erlangen, Friedrich-Alexander-Universität Erlangen-Nürnberg, 91054 Erlangen, Germany; 6 Department of Radiation Oncology, Ordensklinikum Linz, Barmherzige Schwestern, 4010 Linz, Austria; Hans.Geinitz@ordensklinikum.at

**Keywords:** gender, rectal cancer, radiochemotherapy, radiosensitivity, DNA double-strand breaks, radiosensitivity, deposited energy, quality of life, blood values

## Abstract

**Simple Summary:**

In radiotherapy for rectal cancer, the treatment is identical for women and men. In recent years, the question has arisen whether there are gender differences in radiochemotherapy. We have investigated, in detail, differences between men and women, especially with regard to radiation sensitivity. We found no evidence for a difference in radiosensitivity between the sexes. Nevertheless, during radiochemotherapy, women experienced increased impairments in the quality of life, which, however, are restored in the subsequent period. One possibility is an increased sensitivity of women to chemotherapy.

**Abstract:**

Gender is increasingly recognized as an important factor in medicine, although it has long been neglected in medical research in many areas. We have studied the influence of gender in advanced rectal cancer with a special focus on radiosensitivity. For this purpose, we studied a cohort of 495 men (84.1% ≥ T3, 63.6% N1, 17.6%, M1) and 215 women (84.2% ≥ T3, 56.7% N1, 22.8%, M1) who all suffered from advanced rectal cancer and were treated with radiochemotherapy. The energy deposited, DNA double-strand break (dsb) repair, occurrence of chromosomal aberrations, duration of therapy, tumor regression and tumor-infiltrating lymphocytes, laboratory parameters, quality of life and survival were assessed. The residual DNA dsb damage 24 h after irradiation in lymphocytes was identical in both sexes. Furthermore, chromosomal aberrations accurately reflecting radiosensitivity, were similar in both sexes. There were no gender-dependent differences in tumor regression, tumor-infiltrating lymphocytes and outcome indicating no differences in the radiosensitivity of cancer cells. The irradiated tumor volume in women was slightly lower than in men, related to body weight, no difference was observed. However, when the total energy deposited was calculated and related to the body weight, women were exposed to higher amounts of ionizing radiation. During radiochemotherapy, decreases in blood lymphocyte counts and albumin and several quality-of-life parameters such as nausea and vomiting, loss of appetite, and diarrhea were significantly worse in women. There is no difference in radiation sensitivity between men and women in both normal tissue and tumors. During radiochemotherapy, the quality of life deteriorates more in women than in men. However, women also recover quickly and there are no long-term differences in quality of life.

## 1. Introduction

In medicine, gender differences are receiving more and more attention both with regard to the choice of therapy and side effects [1,2]. In oncology, the gender-dependent induction of cancer is also an important topic and clear sex differences are observed. For the vast majority of cancers, men have a significantly increased risk of developing malignancies [3]. In colorectal carcinoma, men have 30% higher incidence rates than women [3]. Causes of colon and rectal cancer seem to be different, and in the case of rectal cancer, alcohol and smoking are clearly of importance as to what might explain the difference. Additionally, there are clear differences depending on the sex with regard to response to cancer therapy and the occurrence of undesirable therapy-related side effects [2,4,5].

From a molecular point of view, several findings support a clear difference in carcinogenesis and therapeutic response. Gender-dependent differences in epigenetic regulation, metabolism, expression of tumor suppressor genes such as p53, cellular senescence, anti-tumor immune reaction and angiogenesis are described [3]. However, the most important differences are probably the hormonal differences and the resulting different effects [6].

Locally advanced rectal cancer is commonly treated with neoadjuvant radiochemotherapy (RCT) or short course radiotherapy alone, followed by total mesorectal excision and adjuvant chemotherapy. New treatment strategies comprise total neoadjuvant treatment or a watch and wait strategy after clinically complete remission following neoadjuvant treatment. RCT carries a certain risk of adverse therapeutic effects both during therapy and in the long term. It is extremely important that acute side effects are well tolerated and do not lead to therapy discontinuation thus comprising oncologic outcome. Long-term therapy consequences can only be experienced if the therapy was successful but might be associated with debilitating symptoms and reduced quality of life. However, judgement on the tolerability of RCT is based on results looking at the average patient population, but does not consider gender [7]. We searched for indicators of differences in men and women in terms of altered efficacy of therapy or side effects. In particular, we were interested as to whether there are gender differences between the radiosensitivity of both normal and tumor tissue and whether this could lead to adverse radiation or chemotherapeutic effects in one sex or an altered tumor response.

## 2. Materials and Methods

### 2.1. Rectal Cancer Cohort and Healthy Individuals

**Figure 1:**
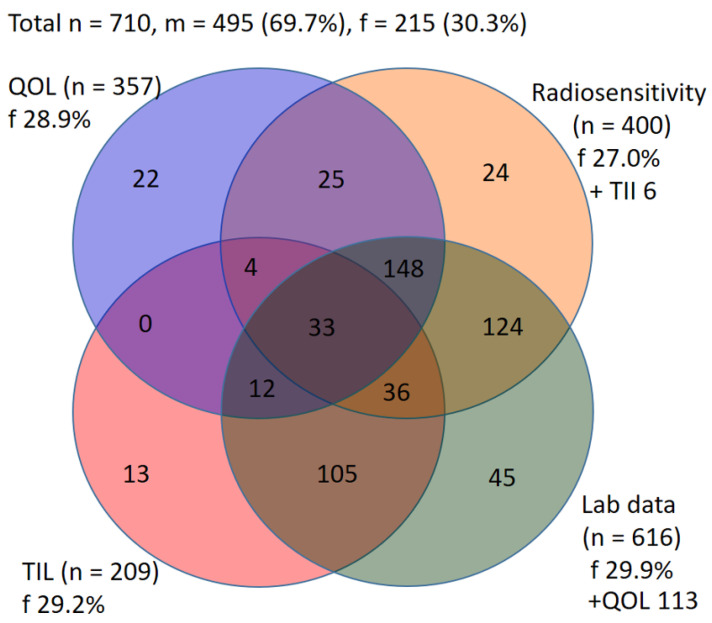
Members of the cohort from four subgroups. The number in the four overlapping circles indicates the number of participants from all four subgroups, corresponding to the three, two and single subgroups. The numbers “+TIL” stand for the intersection of the patient numbers from the radiosensitivity group and the TIL group or “+QOL” for the intersection of the QOL group and the laboratory data group. m = male, f = female, QOL = quality of life cohort, TIL = tumor-infiltrating lymphocytes cohort.

This advanced rectal cancer cohort of 495 males and 215 females was originally derived from three studies on radiosensitivity, tumor-infiltrating lymphocytes and quality of life analyses. The study period was between 2005 and 2018 and represented a consecutive cohort. The fourth cohort of laboratory values is composed of the first three cohorts, including patients who were scheduled for one of the other studies but from whom blood, tissue, or questionnaires were not available and therefore could not be included in these studies. In the radiosensitivity cohort, besides 400 rectal cancer patients, an additional 187 healthy individuals were included as a control group. The γH2AX cohort of 137 rectal cancer patients and 59 healthy individuals was a subgroup of the radiosensitivity cohort. The tumor-infiltrating lymphocytes study consists of 209 patients and the quality of life group consists of 357 patients. Lab data are derived in maximum from 616 patients (Figure 1).

All patients were intended to receive a neoadjuvant RCT consisting of a conventional radiotherapy of 28 fractions with 1.8 Gy each, up to a total dose of 50.4 Gy. Simultaneous chemotherapy was 5-fluorouracil-based. The most commonly used concurrent chemotherapy combination was 5-FU and oxaliplatin. The remaining patients received similar treatment regimens, including 5-FU alone, capecitabine, 5-FU + antibody, 5-FU + cisplatin, or 5-FU + irinotecan. Metastatic patients usually received FOLFOX, FOLFIRI, or FOLFOX-IRI; in some cases, in combination with antibodies. After completion of radiochemotherapy, patients were treated with a total mesorectal resection.

### 2.2. Deposited Energy Calculation

The 95%, 90%, 80%, 60%, 40%, 30% and 20% isodose volumes were derived from the treatment planning software (TPS) Pinnacle (Philips Radiation Oncology Systems, Fitchburg, WI, USA). Deposited energy values were calculated according to the equation described previously (Figure 3B) [8].

### 2.3. γH2AX Detection of DNA Double-Strand Breaks

EDTA blood was drawn before RCT was started and divided into three samples. The background was the initial damage of 0.5 Gy and 30 min repair time and the remaining residual damage 24 h after a dose of 2 Gy IR (Isovolt 160, General electrics, Ahrensburg, Germany). Lymphocytes were then prepared on a slide by cytospin centrifuge and immunostaining with γH2AX (abcam, Cambridge, UK) and counterstained with dapi [9]; 1000 lymphocytes were counted in each group for the average number of foci [10].

### 2.4. Chromosomal Aberrations by Three Color Fluorescence In Situ Hybridization

Before starting the RCT, heparinized blood was drawn and half of it was irradiated with 2 Gy 6-MV ionizing radiation (Oncor, Siemens, Erlangen, Germany) and the other half was taken as background. Lymphocytes were stimulated with phytohemagglutinin and incubated at 37 °C for 48 h. Colcemid was added for 3 h and then the chromosomes were prepared. Chromosomes #1, #2 and #4 were stained with fluorescent probes in red, green and yellow and counterstained with dapi. Metaphases were imaged automatically using a fluorescence microscope (Zeiss, Axioplan 2, Göttingen, Germany) and a specialized software (Metafer 4 V3.10.1, Altlussheim, Germany). An image analysis software (Biomas, Erlangen, Germany) was used to determine the breaks per metaphase. The background value was subtracted from the 2 Gy irradiated values [11,12].

### 2.5. Therapy Duration and Regression Grade

Therapy duration, total dose, and fractions were derived from patient records. The Dworak regression grade was derived from the pathological reports.

### 2.6. Blood Values

Blood values of leucocytes, thrombocytes, monocytes, eosinophils, erythrocytes, hemoglobin, albumin, LDH, creatinine, alkaline phosphatase, C-reactive protein (CRP), glutamic oxaloacetic transaminase (GOT), glutamate pyruvate transaminase (GPT), potassium, thyroid-stimulating hormone and 25-hydroxyvitamin D were obtained from the hospital database. In each case, the value immediately before the start of therapy and before each start of the following therapy weeks was selected. Blood values for thyroid-stimulating hormone and 25-hydroxyvitamin D were only available prior to the start of RCT. 

### 2.7. Tumor-Infiltrating Lymphocytes

Paraffin-embedded tissues from biopsies (*n* = 103) and tumor resections (*n* = 173) were repunched into tissue micro arrays of 2 mm diameter. Immunohistochemical double-staining for FoxP3+ (Ab20034, abcam, Cambridge, UK) and CD8+ (M7103, Agilent, Santa Clara, CA, USA) was performed. Visualization was performed using a Polymer Kit and Fast Red and Polymer Kit and Fast Blue (POLAP-100 Zytomed Systems, Berlin, Germany). Images of each spot were acquired at 400× magnification (Imager Z2, Zeiss, Göttingen, Germany) combined with a Metafer software (Metasystems, Altlussheim, Germany). The number of lymphocytes per square mm were counted separately for tumoral stroma and tumoral epithelium using an image analysis software (Biomas, Erlangen, Germany) [13,14].

### 2.8. Quality of Life

Quality of life was prospectively assessed using the EORTC QLQ C30 and CR38 questionnaires. Time points were before the start of the RCT (*n* = 357), during the RCT at week 2 (*n* = 218) and at the end of the RCT at week 5 (*n* = 195), and after 10 weeks (*n* = 208) immediately prior to surgery. From then on, the questionnaire was answered annually (1 year = 185; 2 years = 105; 3 years = 71; 4 years = 68; 5 years = 48). Scores were calculated according to the official EORTC manual. In the function scales, a higher score means that the patient is doing well in this category. In contrast, a higher score in the symptom category means that the patient has more complaints [15]. Clinical significance for QOL data was assumed at a change of 10% or more.

### 2.9. Survival Curves

Overall survival is calculated as the time from diagnosis to the time of death. Tumor-specific survival was defined as the duration from the date of diagnosis until death due to rectal cancer. Recurrence-free survival was measured from the time of diagnosis to the date of recurrence or death from any cause. Metastasis-free survival was measured from the time of diagnosis to the date of distant metastasis or death from any cause. Local recurrence was defined as recurrent disease within locoregional area and distant recurrence was defined as recurrence beyond the locoregional area. Progression was defined as locoregional failure or distant metastasis. Patients lost to follow-up or who have no events are censored at this time. Median time to follow-up was 49.5 months.

### 2.10. Statistics

All statistical analyses were performed using SPSS version 24 (IBM Inc., Chicago, IL, USA). Student’s *t*-test was used for independent samples and Pearson’s Chi-squared test was used to compare the TNM stages of women and men. Survival plots were generated according to the Kaplan–Meier method [16] and compared using the log-rank test; *p*-values < 0.05 were considered to be statistically significant.

## 3. Results

### 3.1. Patient and Treatment Characteristics

We studied the influence of gender on therapy-related effects in a cohort of 710 patients suffering from rectal cancer; 495 patients were male (69.7%) and 215 female (30.3%). The cohort consisted of four sub-cohorts, namely a cohort testing radiosensitivity (*n* = 400), a quality of live cohort (*n* = 357), a tumor-infiltrating lymphocytes cohort (*n* = 209) and a lab data cohort (*n* = 616). Patients were consecutively included. 

**Figure 2:**
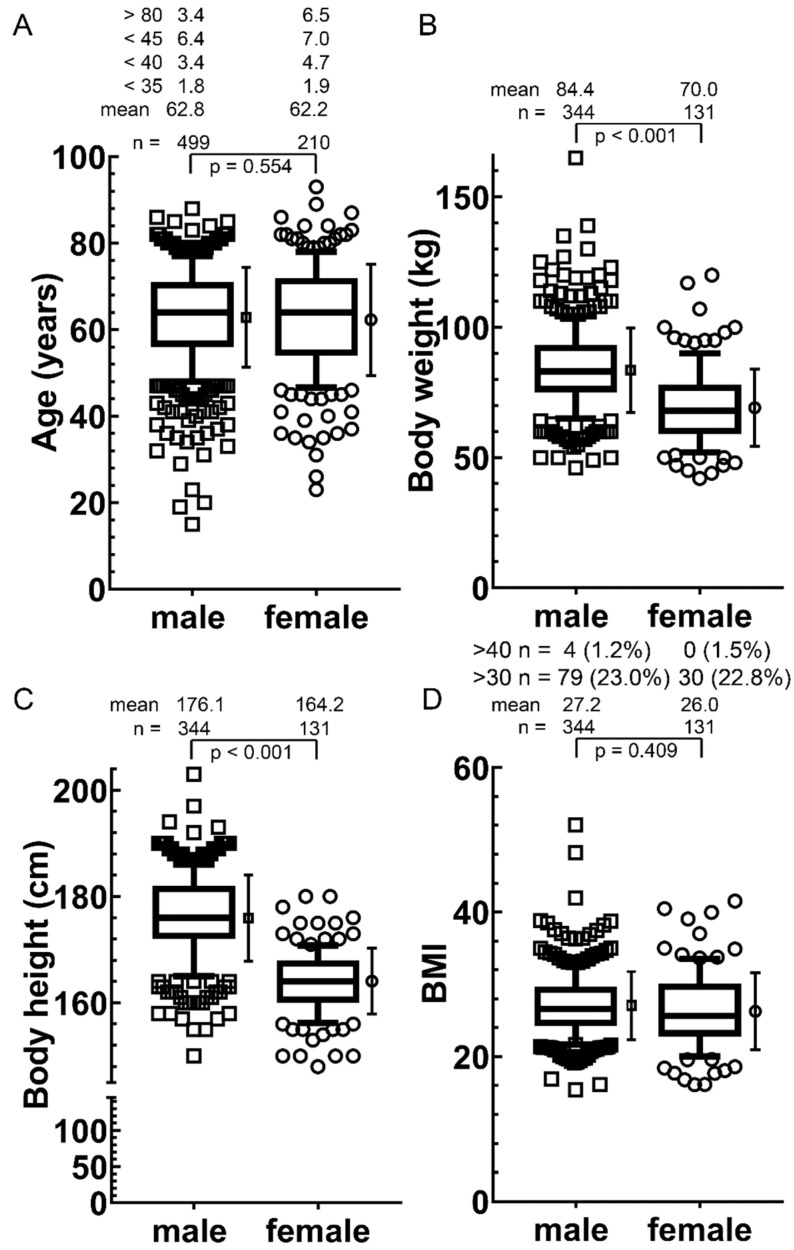
Age, body weight, height and body mass index compared between women and men in the entire cohort. Indicated are the number of individuals: (**A**) Age; (**B**) Body weight; (**C**) Height and (**D**) Body mass index. The box represents the median, the 25th to 75th percentiles and the whiskers of the 10th to 90th percentiles. The mean and standard deviation are shown to the right of the box; *p*-values were calculated by the Student’s *t*-test.

Data of all four endpoints (radiosensitivity, tumor-infiltrating lymphocytes, lab data and quality of life analyses) were available in 33 patients, of three endpoints in 200 patients and of two endpoints in 373 patients (Figure 1). TNM stages were slightly different between females and males (*p* = 0.048) (Table 1). The mean age of female (62.2 years) and male (62.8) patients was nearly identical (*p* = 0.554) (Figure 2A). Body weight (*p* < 0.001) (Figure 2B) and height (*p* < 0.001) (Figure 2C) was significantly higher in males. Nevertheless, the body mass index was comparable (*p* = 0.409) (Figure 2D).

**Figure 3:**
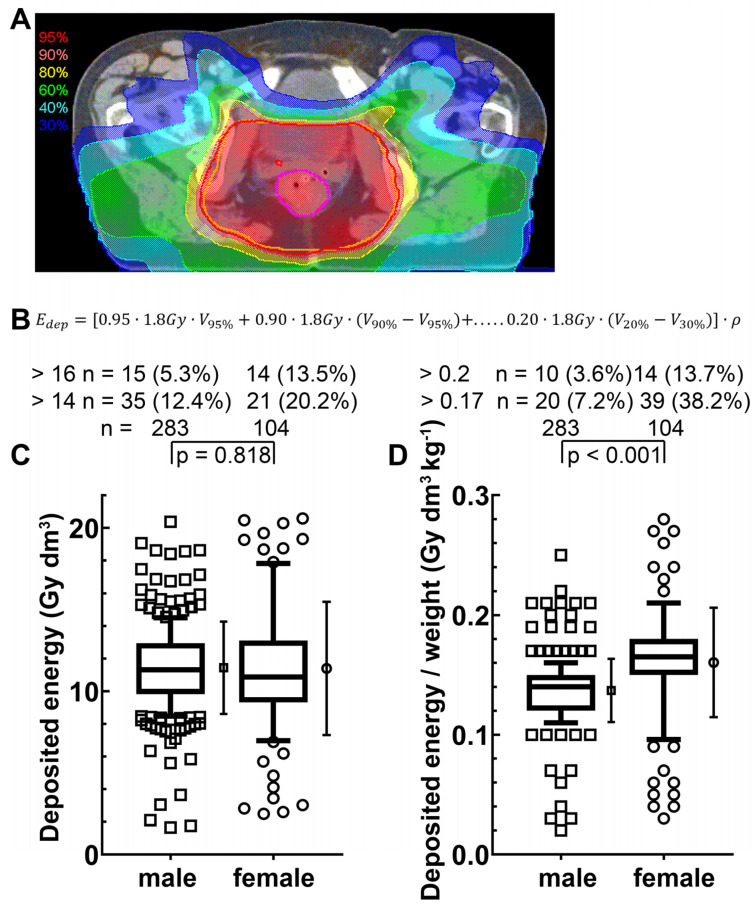
Deposited energy in patients with rectal cancer (radiosensitivity cohort): (**A**) Treatment plan of a patient with rectal cancer where the isodose ranges are marked with different color wash. The magenta line represents the gross target volume; the inner yellow line represents the planning target volume; (**B**) The deposited energy (Edep) is calculated using the equation given, where V is the volume and ρ is the density; (**C**) The deposited energy and (**D**) The deposited energy related to body mass in the cohort. The box represents the 25th to 75th percentiles and the whiskers represent the 10th to 90th percentiles. The mean and standard deviation are shown to the right of the box; *p*- values were calculated by the Student’s *t*-test.

We assessed whether there was a difference in the total deposited energy between males and females. We used the isodose volumes of the radiation planning system and calculated a deposited energy of each patient (Figure 3A). The deposited energy is defined as the sum of the isodose dose values multiplied by the volume of this dose level and the mass density (Figure 3B). Equal amounts of energy were deposited in males and females (Figure 3C). However, taking into account that females have a lower mass, the mean dose related to the total body is significantly higher in females (*p* < 0.001) (Figure 3D). The 95% isodose volume to treat the tumor, however, tended to be smaller in women than in men (*p* = 0.088). In terms of body weight, there was no difference, indicating that the tumor region was treated equally in men and women (*p* = 0.488), as shown in Supplementary Figure S1. Chemotherapy given simultaneously was 5-FU based in males in 90.9% and in females in 85.6% of the cases. The healthy control cohort consisted of 79 men and 108 women with mean ages of 51 years and 49.4 years, respectively.

### 3.2. DNA Double-Strand Breaks and Chromosomal Aberrations

**Figure 4:**
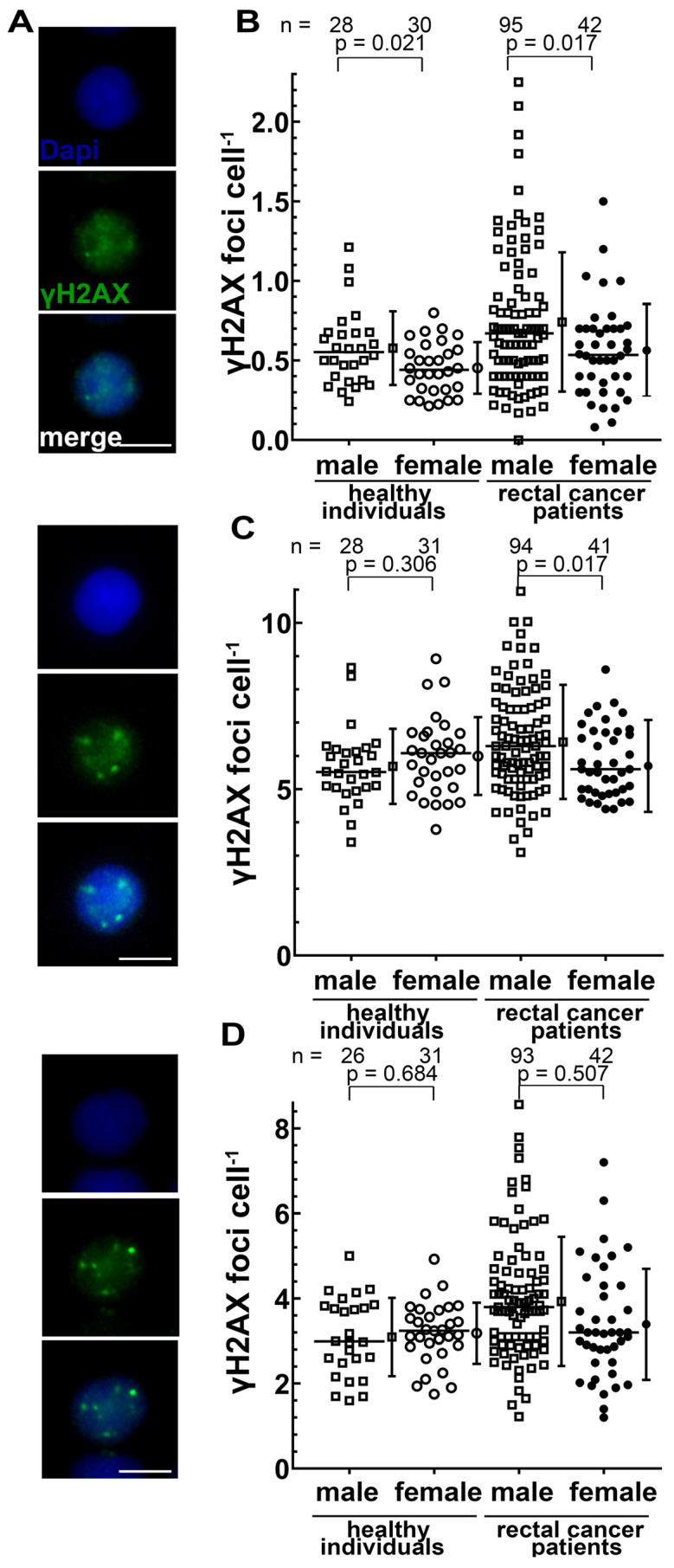
Number of DNA double-strand breaks per lymphocyte quantified by γH2AX after immunostaining of lymphocytes from healthy individuals and patients with rectal cancer (radiosensitivity cohort): (**A**) Representative images of an unirradiated cell, a 0.5 Gy irradiated cell with 30 min and a 2 Gy irradiated cell with 24h repair time. Blue staining is dapi and green staining is γH2AX; (**B**) Pre-existing γH2AX foci; (**C**) Initially formed γH2AX foci 30 min after an IR dose of 0.5 Gy and (**D**) γH2AX foci after an IR dose of 2 Gy and a repair time of 24 h. The mean and standard deviation are shown to the right of the dot plots; *p*-values were calculated by the Student’s *t*-test.

A key question of our analyses was whether a gender difference in the DNA double- strand break (dsb) repair and chromosomal aberrations induction exists. DNA dsb were analyzed by γH2AX staining (Figure 4A) in blood lymphocytes obtained prior to RCT. DNA dsb background rates in men were slightly higher in both healthy individuals (*p* = 0.021) and patients with rectal cancer (*p* = 0.017) (Figure 4B). The initial dsbs 30 min after an ex vivo dose of 0.5 Gy ionizing radiation were equal in healthy individuals and slightly higher in males with rectal cancer (Figure 4C). The remaining DNA DSB after a dose of 2 Gy and 24 h of repair time were identical between genders in healthy subjects (*p* = 0.684) and patients with rectal cancer (*p* = 0.507) (Figure 4D).

**Figure 5:**
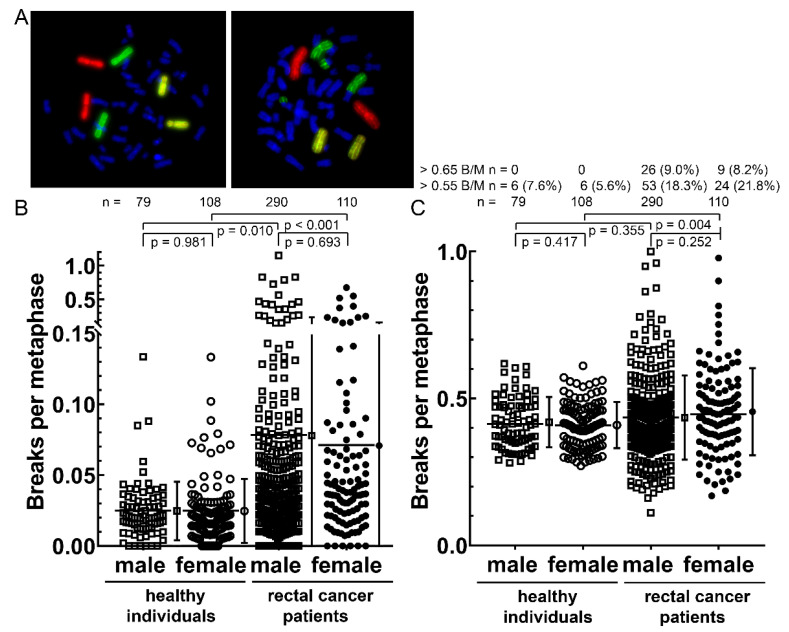
Radiosensitivity testing by three color fluorescence in situ hybridization of chromosomes #1, #2 and #4 (radiosensitivity cohort and healthy individuals). Metaphase spreads of human blood lymphocytes with chromosomes #1 (red), #2 (green) and #4 (yellow) stained. DNA was counterstained with DAPI (blue): (**A**) Normal metaphase spread in comparison to a metaphase spread with a translocation and a dicentric aberration each in chromosome #2, in sum scored with 4 breaks. Chromosomal aberrations in 179 healthy individuals and 400 patients suffering from rectal cancer; (**B**) Individual background B/M rates for both cohorts; (**C**) After ex vivo IR of 2 Gy. The mean and standard deviation are shown to the right of the dot plots; *p*-values were calculated by the Student’s *t*-test.

Chromosomal aberrations were analyzed by three color fluorescence in situ hybridization (Figure 5A). Background levels of both genders in rectal cancer patients were clearly higher compared to healthy individuals, yet between genders no difference was observed (Figure 5B). After 2 Gy ex vivo irradiation, there was no difference in between healthy individuals and rectal cancer patients between sexes. The same was observed when only stable or unstable or complex aberrations were compared (Supplementary Figure S2A–C).

### 3.3. Total Treatment Time, Tumor Regression, Blood Counts and Serum Parameters

**Figure 6:**
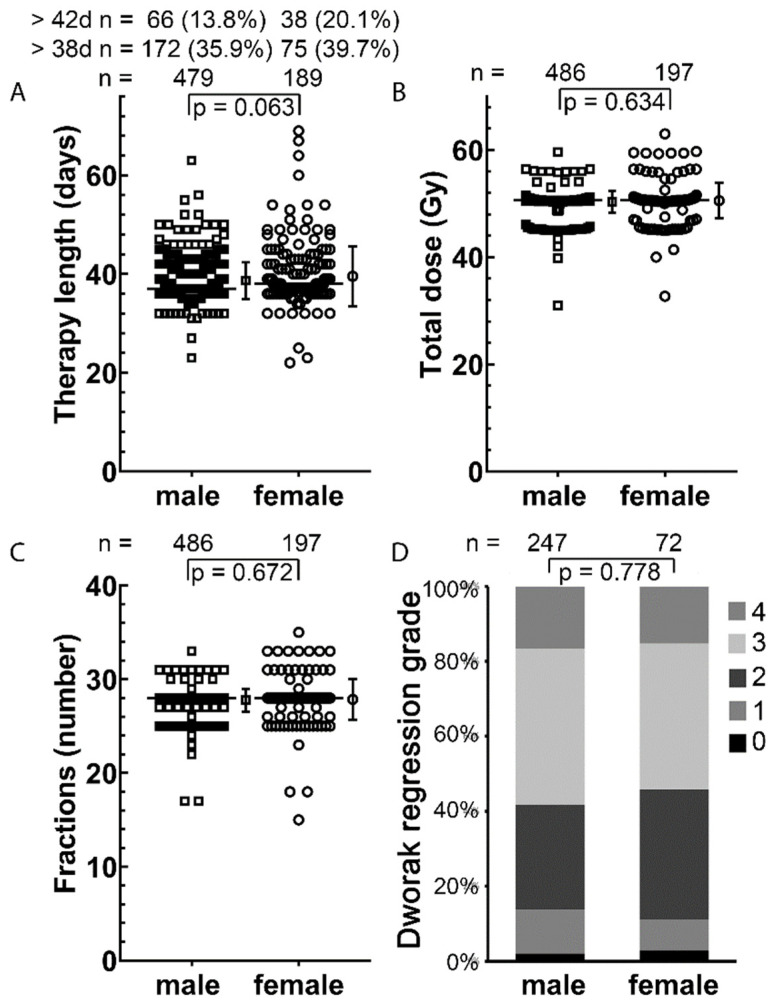
Therapy length, total dose and number of fractions of the entire cohort: (**A**) Length of therapy in days of 479 men and 189 women; (**B**) Total dose applied in Gy; (**C**) Number of fractions irradiated. The line indicates the median; (**D**) Regression of the cancer cells after RCT of the radiosensitivity cohort. Dworak regression grade means 0 is no regression and 4 is no remaining cancer cells. The mean and standard deviation are shown to the right of the dot plots; *p*-values were calculated by the Student’s *t*-test and the Fisher’s exact test were used for regression.

During radiotherapy, higher energy per mass was deposited in females. In addition, there was only marginal difference in DNA DSB repair and chromosomal aberrations. Therefore we examined indicators of higher toxicity. An interruption of RCT and thus a prolongation of total radiation treatment time can be an indicator of a stronger toxic effect of the RCT. Therapy length was slightly longer by nearly one day in women as compared to men (38.4 day versus 37.8day, *p* = 0.063) (Figure 6A). Total dose (49.9 Gy versus 49.2 Gy *p* = 0.634) and fractions (27.5 versus 26.8 *p* = 0.672) were comparable between both genders (Figure 6B,C). An indicator of radiation sensitivity is histological tumor regression after RCT. For this purpose, Dworak grading was used that indicated a mean value of 2.59 for men and 2.56 for women, which did not show a significant difference (*p* = 0.778) (Figure 6D). 

**Figure 7:**
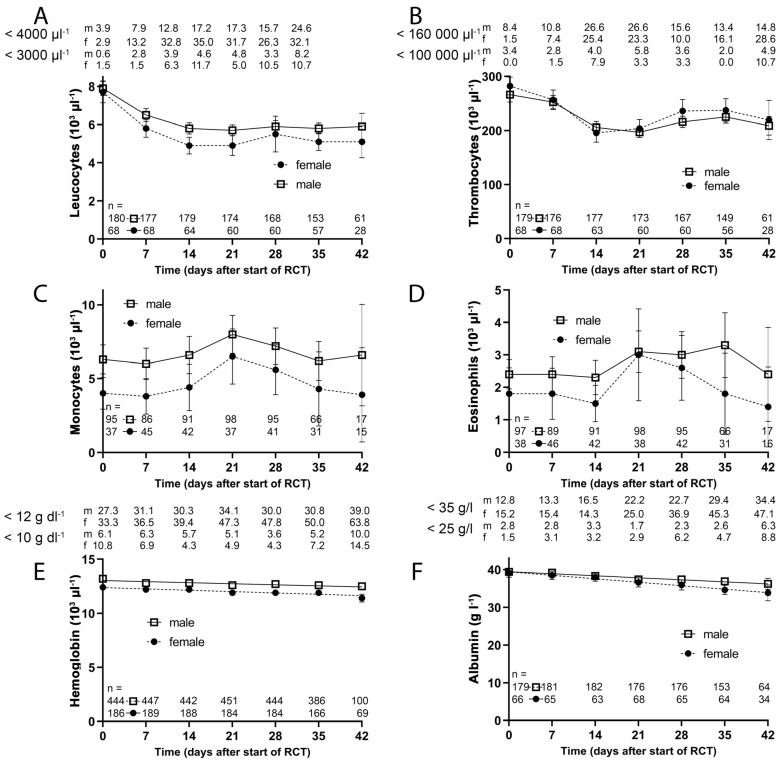
Blood values prior to and during the RCT of the lab data cohort. The filled dots represent women and the open rectangles represent men. The number of people with blood samples is indicated. The number of (**A**) Leukocytes; (**B**) Platelets; (**C**) Monocytes; (**D**) Eosinophils and the amount of (**E**) hemoglobin and (**F**) Albumin were given. Length of the error bars is the 95% confidence interval for the mean. m = male, f = female.

Changes in leukocyte blood counts may indicate differences in toxicity and were studied during RCT. Leucocyte decrease was mildly enhanced in females compared to males, while thrombocytes, monocytes, eosinophils (Figure 7A–D) and erythrocytes did not differ between the two groups. Hemoglobin decreased constantly in both groups (Figure 7E) while albumin decreased slightly more in females (Figure 7F). There was no gender specific difference in thyroid-stimulating hormone prior to therapy (*p* = 0.537), yet women had slightly higher levels in 25-hydroxyvitamin D (*p* = 0.043). Erythrocytes, LDH, creatinine, alkaline phosphatase, CRP, GOT, GPT and potassium did not clearly differ during RCT between both genders (Supplementary Figures S3–S5).

### 3.4. Tumor-Infiltrating Lymphocytes

**Figure 8:**
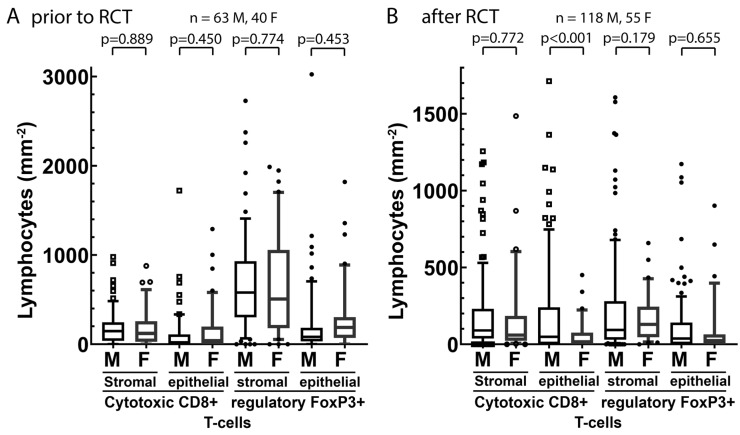
Stromal and intraepithelial cell density distributions of CD8+ and FoxP3+ regulatory T cells from samples (**A**) prior to RCT and, on average, (**B**) 55 days after RCT (tumor-infiltrating lymphocytes cohort). The center line represents the median value, while the box indicates the interquartile range (IQR). The whiskers represent 1.5 times the IQR or the minimum/maximum. Outliers are indicated by symbols; *p*-values were calculated by the Student’s *t*-test. The box represents the 25th to 75th percentiles and the whiskers represent the 10th to 90th percentiles. M = male, F = female, RCT = radiochemotherapy.

The immune response against the tumor could differ depending on sex, so that tumor-infiltrating CD8+ cytotoxic lymphocytes and FoxP3+ regulatory T lymphocytes prior to RCT in the biopsy and about 55 days after RCT in the surgical specimen were compared between sexes. Lymphocyte counts were analyzed in the stromal and epithelial compartment of the tumors. No differences were found between genders with the exception of lower counts of CD8+ cytotoxic lymphocytes in the epithelial compartment after RCT in women (Figure 8).

### 3.5. Health-Related Quality of Life

**Figure 9:**
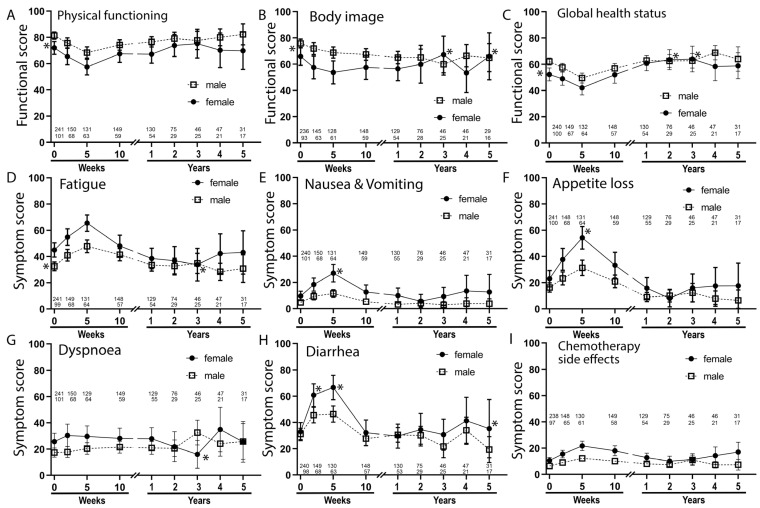
Quality of life by QLQ C30 and CR38 questionnaires of the quality of life cohort. Data points are baseline, during RCT (2nd and 3rd point), directly prior to surgery (4th point) and 1 to 5 years after beginning of RCT. Functional scores (**A**–**C**) and symptom scores (**D**–**I**). The scores are: (**A**) Physical functioning (**B**) Body image (**C**) Global health status (**D**) Fatigue (**E**) Nausea & Vomiting (**F**) Appetite loss (**G**) Dyspnoea (**H**) Diarrhea (**I**) Chemotherapy side effects. Asterisks to the left of the abscissa mark differences of more than 10% in the baseline, and asterisks (*) in the time data mark a change of more than 10 percentage points from the baseline. The length of the error bars corresponds to the 95% confidence interval for the mean. The number of patients who answered the questionnaires is given.

Furthermore, we analyzed quality of life to study therapy-related side effects during therapy and for a long follow-up period of up to 5 years after RCT. The QLQ-C30 and C38 questionnaires were used. In nearly all function and symptom scores, the females’ baselines tended to be worse or were 10% points below the males’ scores and were therefore regarded as significantly inferior. During RCT, females tend to deteriorate more than males in several scores. This was most pronounced for nausea and vomiting, appetite loss and diarrhea (Figure 9). However, already ten weeks after the beginning of RCT, most symptoms returned to baseline. One year after beginning the RCT or later, there was even an improvement in the state of women compared to men of 10% or more with regard to body image, fatigue, dyspnea and diarrhea. This was true for most of the other functional scores (Supplementary Figures S6–S9).

### 3.6. Survival and Oncologic Outcome

**Figure 10:**
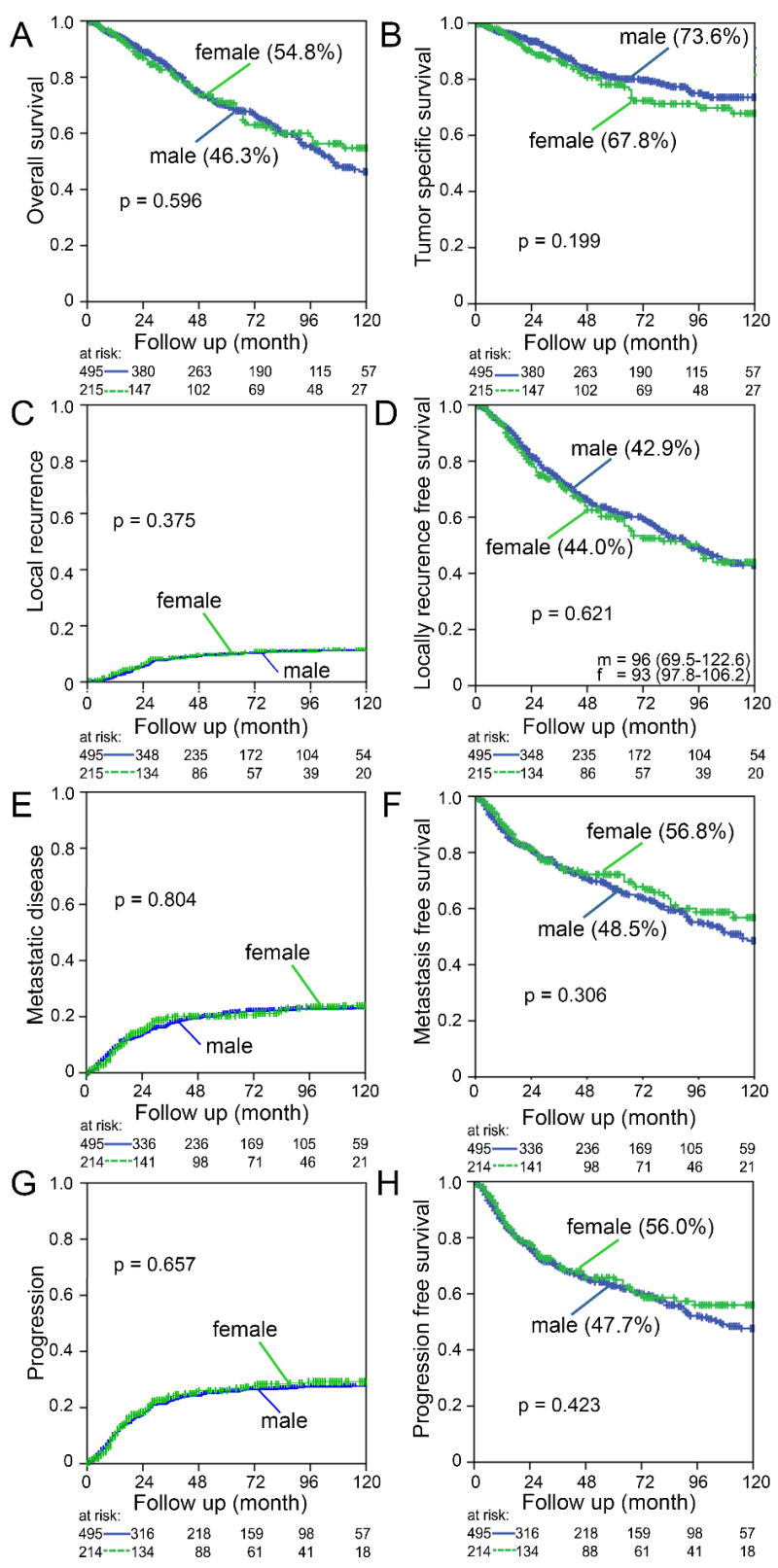
Ten year follow-up of male and female patients suffering from rectal cancer of the entire cohort: (**A**) Overall survival; (**B**) Tumor-specific survival; (**C**) Cumulative incidence of local recurrence; (**D**) Local recurrence-free survival; (**E**) Cumulative incidence of metastatic disease; (**F**) Metastasis-free survival; (**G**) Cumulative incidence of any recurrence and (**H**) Progression-free survival. The 10-year survival is given in brackets. Log-rank test was used to calculate *p*-values.

Finally, we analyzed the difference in survival between men and women. We found no differences in overall survival (*p* = 0.596), tumor-specific survival (*p* = 0.199), local recurrence free survival (*p* = 0.621), distant metastasis free survival (*p* = 0.306) or progression- free survival (*p* = 0.423) (Figure 10). Cumulative incidence of local recurrence (*p* = 0.375), metastatic disease (*p* = 0.804) and progression (*p* = 0.657) was not different between men and women. 

## 4. Discussion

The most significant finding of this study was an only minor difference in DNA repair between genders in normal tissue but no difference in the occurrence of chromosomal aberrations. This data were derived from a large number (*n* = 587) of rectal cancer patients and healthy individuals. In the repair examined with γH2AX, the accuracy of the repair plays an important role in addition to the reconnection, but this can only be checked to a limited extent with γH2AX [17]. In contrast, chromosomal aberrations reflect not only the repair but also the mutation frequency and thus, to a certain extent, the correctness of the repair. Additionally, chromosomal aberrations reflect individual radiosensitivity very well [18]. Therefore, this finding reveals that DNA repair and DNA damage processing is not different between males and females. Since there is a notion that instable aberrations predominantly reflect the occurrence of cell death and thus side effects, and stable aberrations represent more the stochastic risk [19], these parameters were also studied. There were no identifiable differences between both sexes. In epidemiological observations from the Hiroshima and Nagasaki atomic bomb studies, the stochastic risk of cancer development was significantly higher in women [20]. In the context of our data, this would suggest that the increased stochastic risk of the Hiroshima and Nagasaki cohort is not indicative of poorer repair or damage processing, but later processes of carcinogenesis where hormonal differences then lead to the development of cancer [3]. 

On the contrary, in a study with fibroblast cell lines from 89 women and 63 men, a higher sensitivity of the female cell lines was found despite a very strong variation in the colony-forming assay. Likewise, significant gender differences were found in 10 radiation responsive genes [21]. A review on Sex Difference of Radiation Response reports that long-term radiosensitivity in females is higher than that in males. The same paper also states that data are still insufficient [22].

To investigate the radiation sensitivity of the tumor, we measured the regression of tumor cells after RCT in surgical tumor specimens, on average, 55 days after the end of RCT. We found no sex-dependent difference in tumor regression grades. In addition, no differences in the amount of tumor-infiltrating CD8+ and FoxP3+ lymphocytes were detected. This indicates that there is no differential immune response between the sexes in these two lymphocyte types. Finally, no difference in the therapy outcome was found. This also indicates that the radiosensitivity of the tumor is not different between males and females.

The clearest difference between men and women during the RCT was seen in quality of life. Already at baseline, women often had worse scores than men. There was a general trend for these scores to worsen during therapy and to improve significantly after RCT. In long-term follow-up, even an improved state in women compared to men as opposed to the baseline was shown. This suggests an increased acute sensitivity to RCT. It is not clear whether this is more an effect of radiotherapy or chemotherapy. Most blood parameters proceeded similarly between sexes. However, leukocytes and albumin decrease more in women than in men.

One possible explanation for these effects, as we have shown, could be the relatively higher deposited energy of radiation in women. Although the 95% isodose volume is smaller in women than in men, no difference can be seen in relation to body weight. If the total deposited energy is calculated in relation to body weight, then 17% more energy per mass is deposited in women. The reason for this is probably the different anatomical situation in women compared to men. Since the BMI was the same in both groups, overweightness in women cannot be the reason for this difference. Toxicity of chemotherapeutic agents could be an alternative explanation for the increased side effects in women. Women have an increased risk of therapy-related side effects after application of chemotherapeutic agents [23,24]. This has been reported for colorectal cancer [25] and rectal cancer [26]. Fluorouracil as the main component of therapy causes higher toxicity in women [27]. This could be due to the lower concentration of the 5-FU degrading enzyme dihydropyridine dehydrogenase [2] or more frequent polymorphisms in women’s enzyme [28,29]. Moreover, the gender-specific heterogeneous body fat composition could have an influence [26,29]. In addition, the calculation of body surface area in women leads to a relatively high dose, since the percentage of fat in women is higher [30]. A combination of both the increased deposited energy per mass and the increased side effects of chemotherapy might also be operational. It was suggested to use lean body mass instead of body surface area to calculate the 5-FU dose [30].

In general, increased side effects in women were limited to the duration of therapy and disappeared in the long-run. In terms of quality of life, women tended to achieve better values in the long-term. Finally, there was no observed difference between men and women in the incidence of local recurrences or distant metastases, or in progression-free, tumor-specific, or overall survival. This is in line with multiple observations of no difference between men and women in survival parameters [26,31].

## 5. Conclusions

Radiation sensitivity of normal tissue and tumor appears to be the same in males and females in this large set of rectal cancer patients and healthy individuals. During radiochemotherapy, QoL deteriorates more in women than in men, and women also experience larger depletions in leucocyte counts during treatment. We postulate that increased chemotherapy-derived toxicity and a slightly higher deposited energy is the underlying cause for these phenomena. 

## Figures and Tables

**Table 1:** Tumor stage according to gender of the entire cohort.

Stage		Male (%)	Female (%)	Significance (*p*)
cT-stage	1	13 (2.6%)	8 (3.7%)	
	2	66 (13.3%)	26 (12.1%)	
	3	332 (67.1%)	127 (59.1%)	
	4	84 (17.0%)	54 (25.1%)	0.011
pN-stage	0	180 (36.4%)	93 (43.3%)	
	1	315 (63.6%)	122 (56.7%)	0.093
cM-stage	0	408 (82.4%)	166 (77.2%)	
	1	87 (17.6%)	49 (22.8%)	0.144

Significance calculated by Pearson’s Chi-squared test. For the cT-stages, only stage 3 and 4 were used for the statistical calculation.

## Data Availability

The datasets used and/or analyzed during the current study are available from the corresponding author on reasonable request.

## Supplementary Materials

The following supporting information can be downloaded at: https://www.mdpi.com/article/10.3390/cancers14010148/s1, Figure S1: Irradiated volume of the radiosensitivity cohort; Figure S2: Different types of chromosomal aberrations in females and males of the radiosensitivity cohort; Figure S3: Blood values prior to and during the RCT; Figure S4: Blood values prior to and during the RCT; Figure S5: Blood values prior to and during the RCT; Figure S6: Functional scores of quality of life by QLQ C30 questionnaires; Figure S7: Functional scores of quality of life by QLQ CR38 questionnaires; Figure S8: Symptom scores of quality of life by QLQ C30 questionnaires; Figure S9: Symptom scores of quality of life by QLQ C30 questionnaires.
