# Supplementary material for: Influence of Gender on Radiosensitivity during Radiochemotherapy of Advanced Rectal Cancer
Source: Cancers (Basel). 2021 Dec 29;14(1):148. doi: 10.3390/cancers14010148 (PMC8750676; doi:10.3390/cancers14010148)
Supplement: Supplementary file 1 [file cancers-14-00148-s001.zip › cancers-1505432-supplementary.pdf]

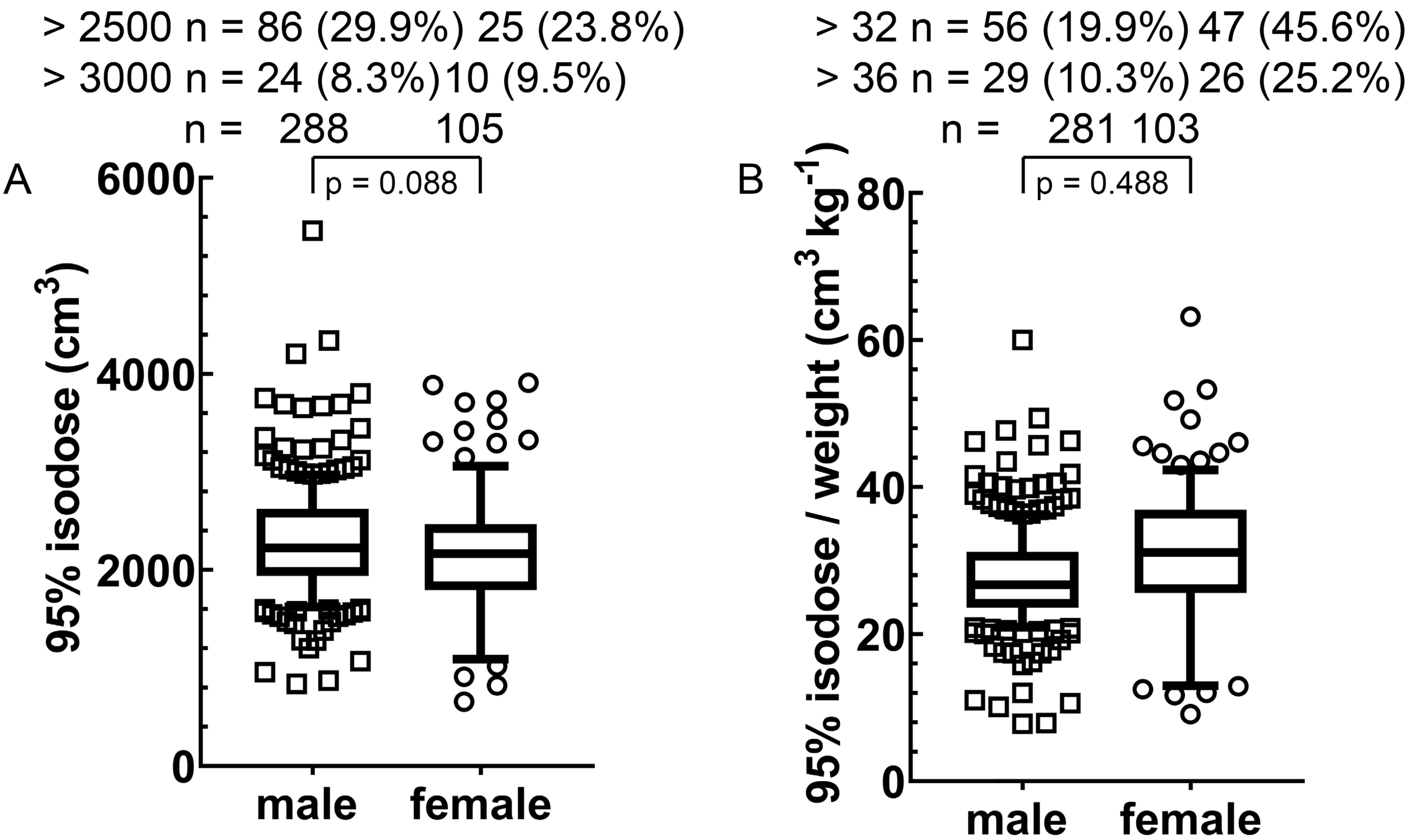

Supplementary Figure S1: Irradiated volume of the radiosensitivity cohort. The 95% isodose volume describes the range in which at least 95% of the prescribed dose is deposited and which corresponds to the planning target volume. The box represents the 25th to 75th percentiles and the whiskers the 10th to 90th percentiles. p values were calculated by the Student's t-test.

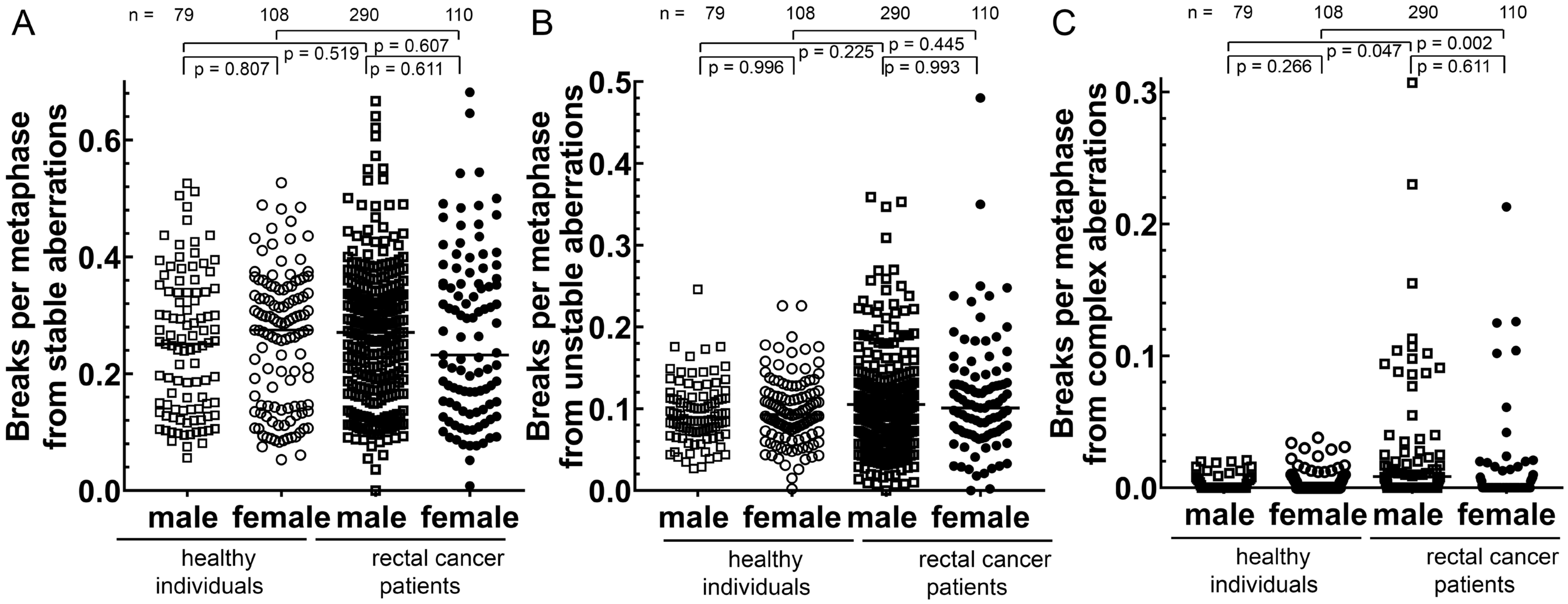

Supplementary Figure S2: Different types of chromosomal aberrations in females and males of the radiosensitivity cohort. (A) Breaks per metaphase that are solely related to stable aberrations such as translocations and insertions. (B) Breaks derived from unstable aberration like dicentrics, rings and breaks. (C) Breaks derived from complex aberrations including aberrations with more than three breaks in one chromosome. Aberrations were analyzed after ex vivo IR of 2Gy. p values were calculated by the Student's t-test.

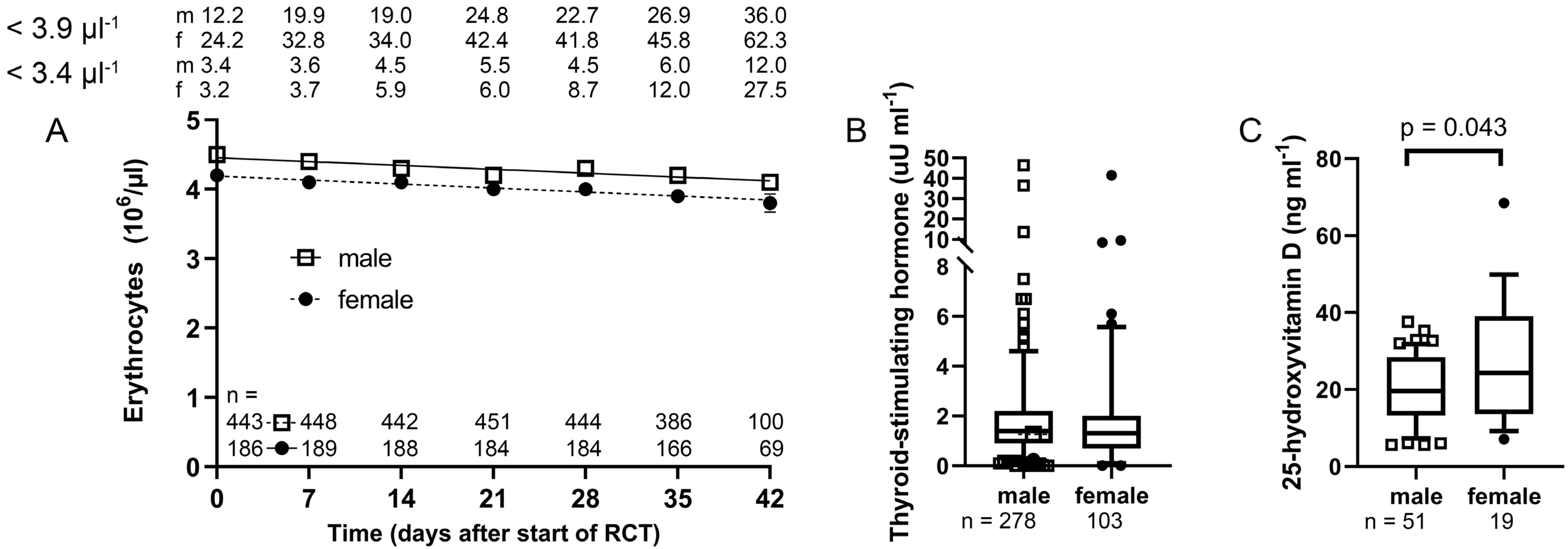

Supplementary Figure S3: Blood values prior to and during the RCT (Lab data cohort). Filled dots represent women and the open rectangles represent men. The number of individuals with blood samples is indicated. (A) The number of erytrocytes. (B) The amount of thyroid-stimulating hormone and (C) 25-hydroxyvitamin prior to RCT were given. Length of the error bars is the 95% confidence interval for the mean.

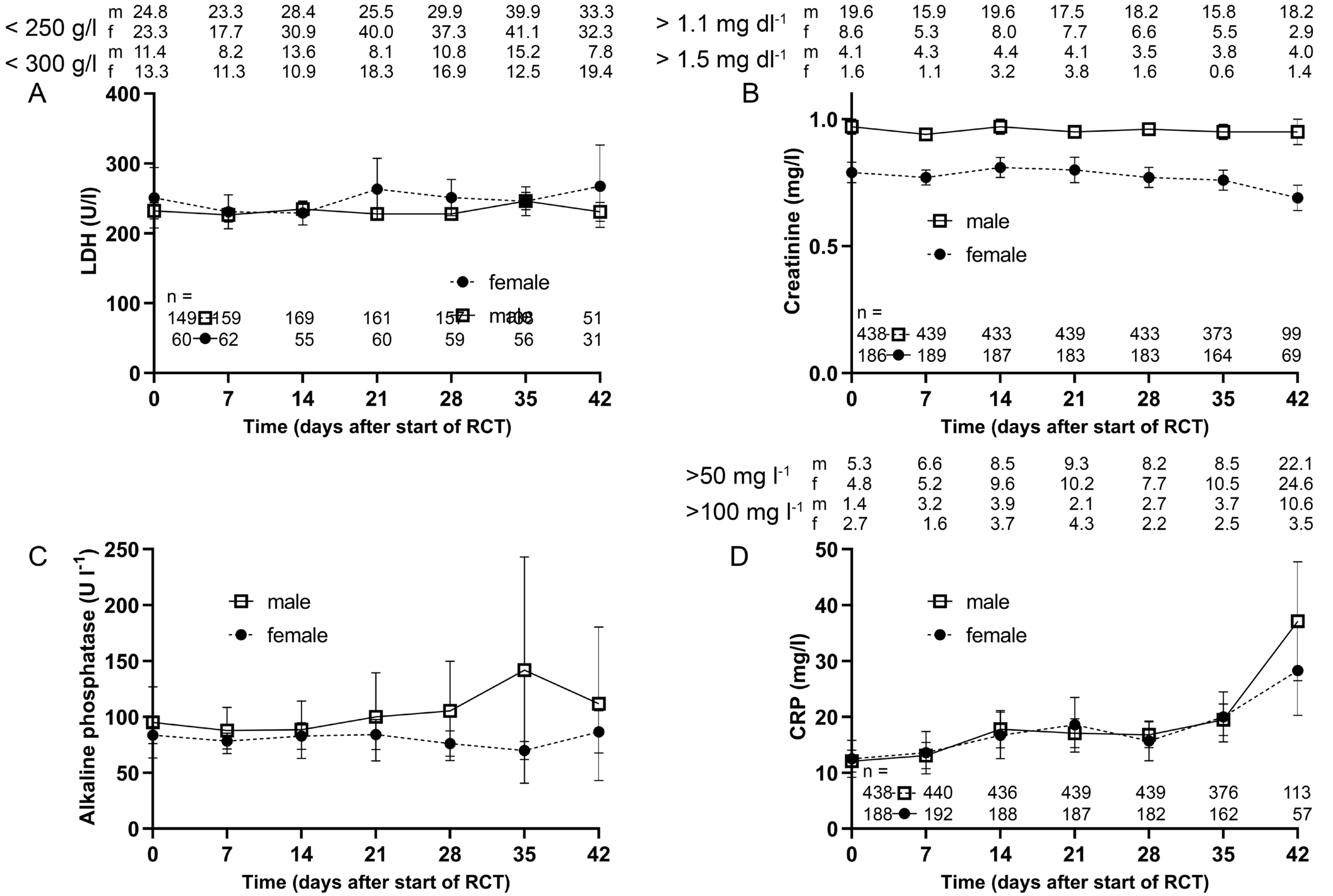

Supplementary Figure S4: Blood values prior to and during the RCT (Lab data cohort). Filled dots represent women and the open rectangles represent men. The number of individuals with blood samples is indicated. The amount of (A) LDH, (B) creatinine (C) alkaline phosphatase and (D) CRP were studied . Length of the error bars is the 95% confidence interval for the mean.

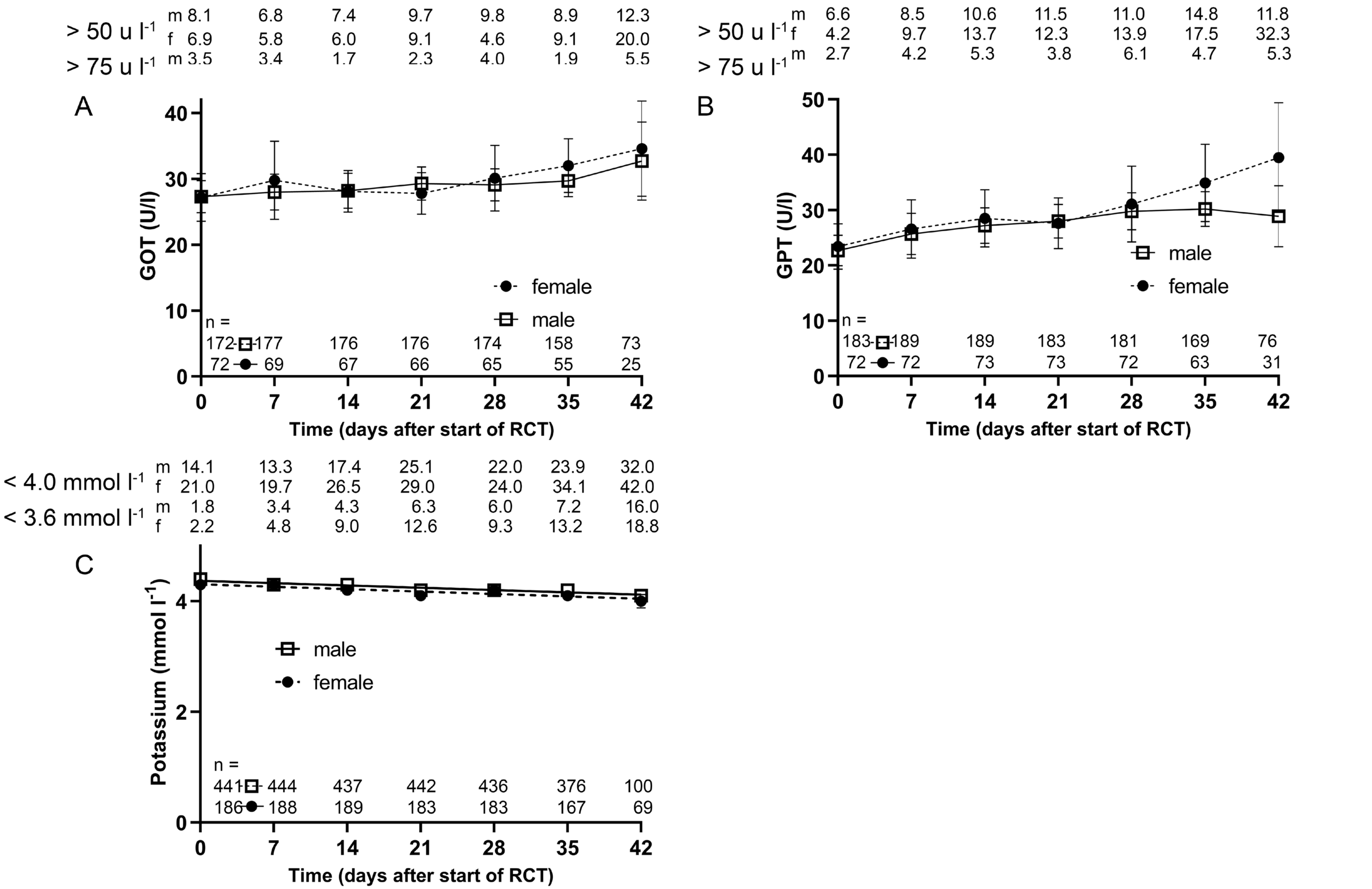

Supplementary Figure S5: Blood values prior to and during the RCT (Lab data cohort). Filled dots represent women and the open rectangles represent men. The number of individuals with blood samples is indicated. The amount of (A) GOT, (B) GPT and (C) potassium were studied. Length of the error bars is the 95% confidence interval for the mean.

Functional scores QLQ-C30

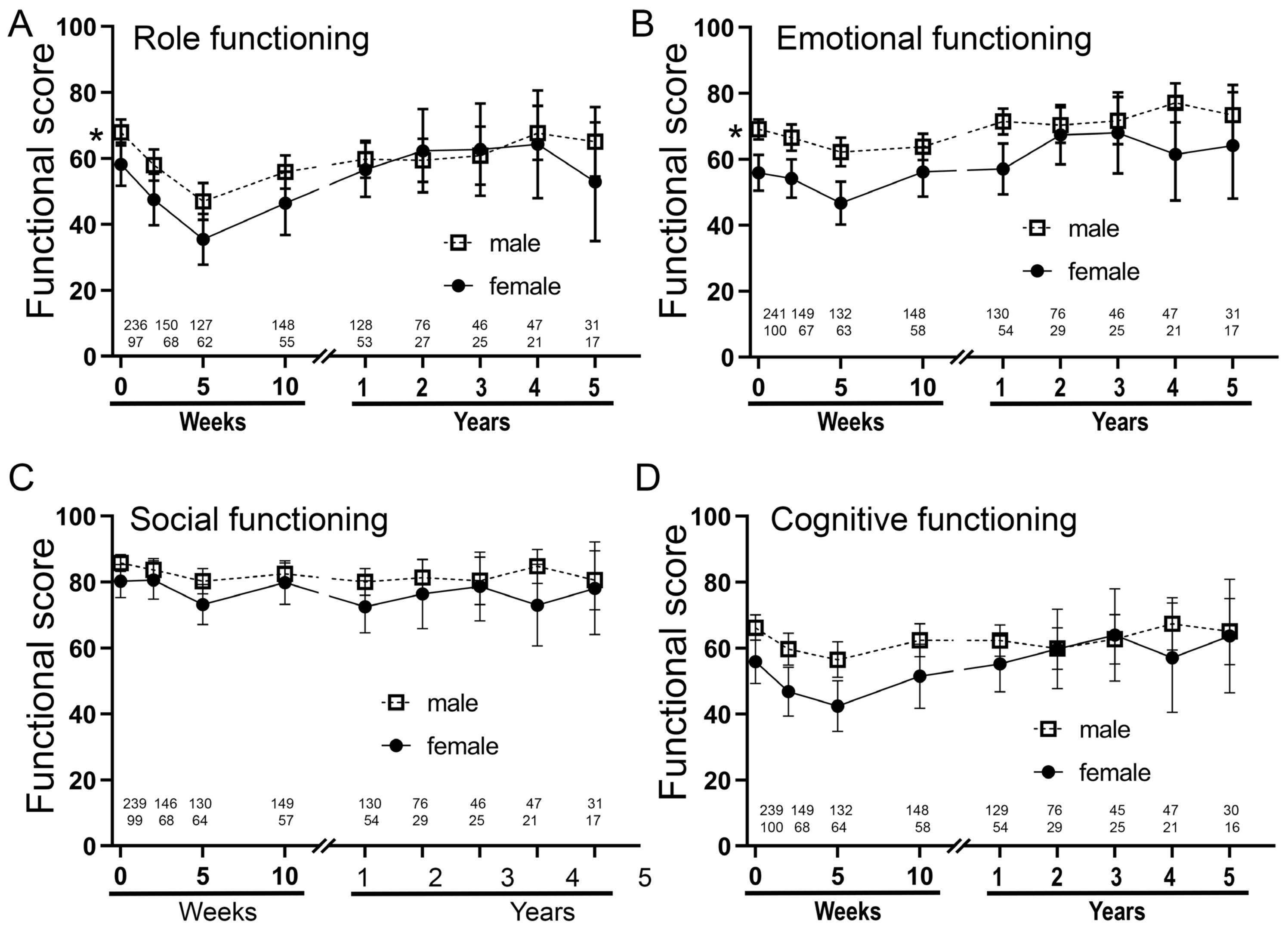

Supplementary Figure S6: Functional scores of quality of life by QLQ C30 questionnaires (Quality of live cohort). Data points are baseline, during RCT (2nd and 3rd point), directly prior to surgery (4th point) and 1 to 5 years after beginning of RCT. (A) Role functioning (B) emotional functioning (C) social functioning and (D) cognitive functioning. Asterisks to the left of the abscissa mark differences of more than 10% in the baseline and asterisks in the time data mark a change of more than 10 percentage points from the baseline. The length of the error bars corresponds to the 95% confidence interval for the mean. The number of patients who answered the questionnaires is given.

Functional scores QLQ CR38

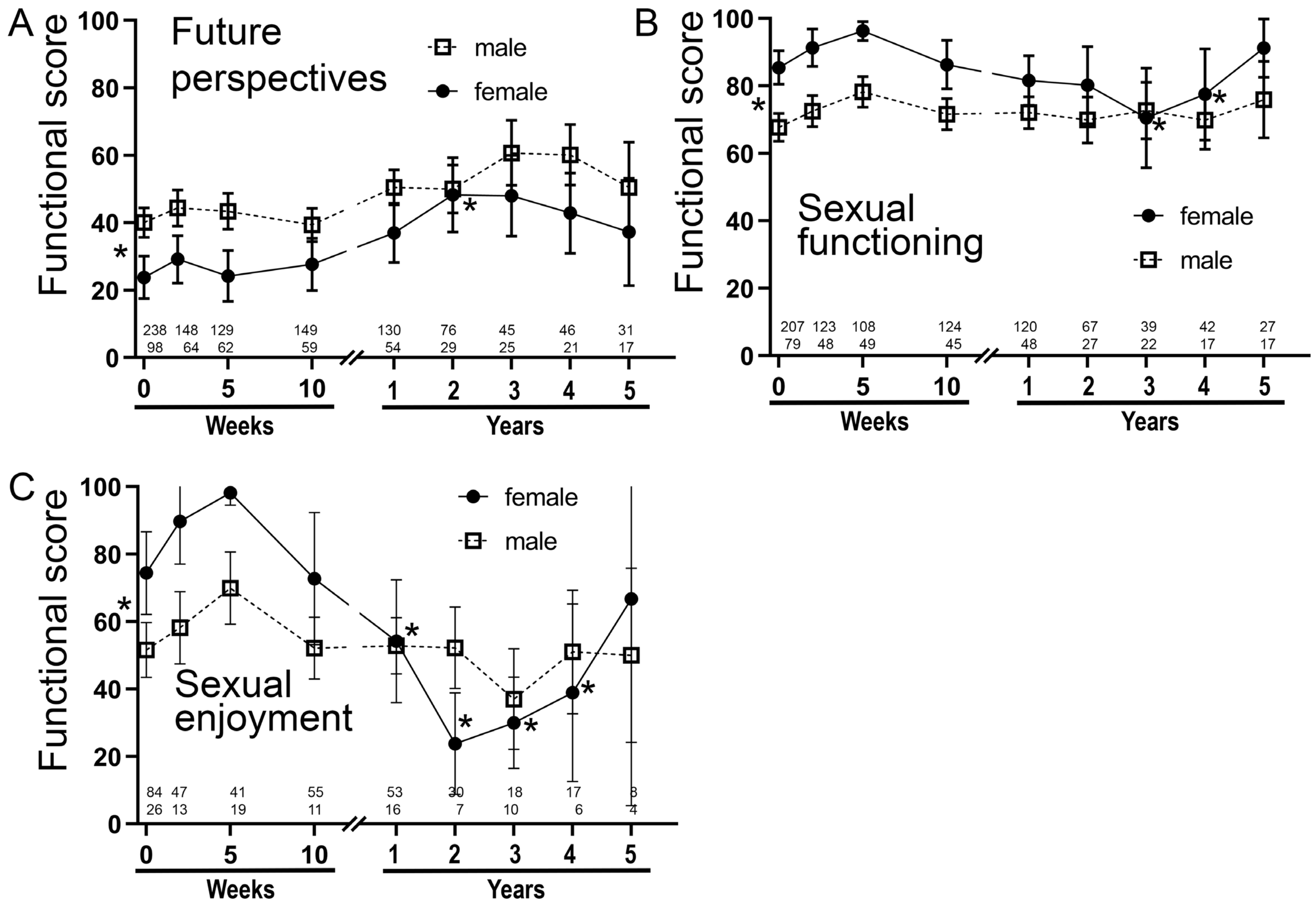

Supplementary Figure S7: Functional scores of quality of life by QLQ CR38 questionnaires (Quality of live cohort). Data points are baseline, during RCT (2nd and 3rd point), directly prior to surgery (4th point) and 1 to 5 years after beginning of RCT. (A) Future perspectives (B) Sexual functioning and (C) sexual enjoyment. Asterisks to the left of the abscissa mark differences of more than 10% in the baseline and asterisks in the time data mark a change of more than 10 percentage points from the baseline. The length of the error bars corresponds to the 95% confidence interval for the mean. The number of patients who answered the questionnaires is given.

Symptome scores QLQ-C30

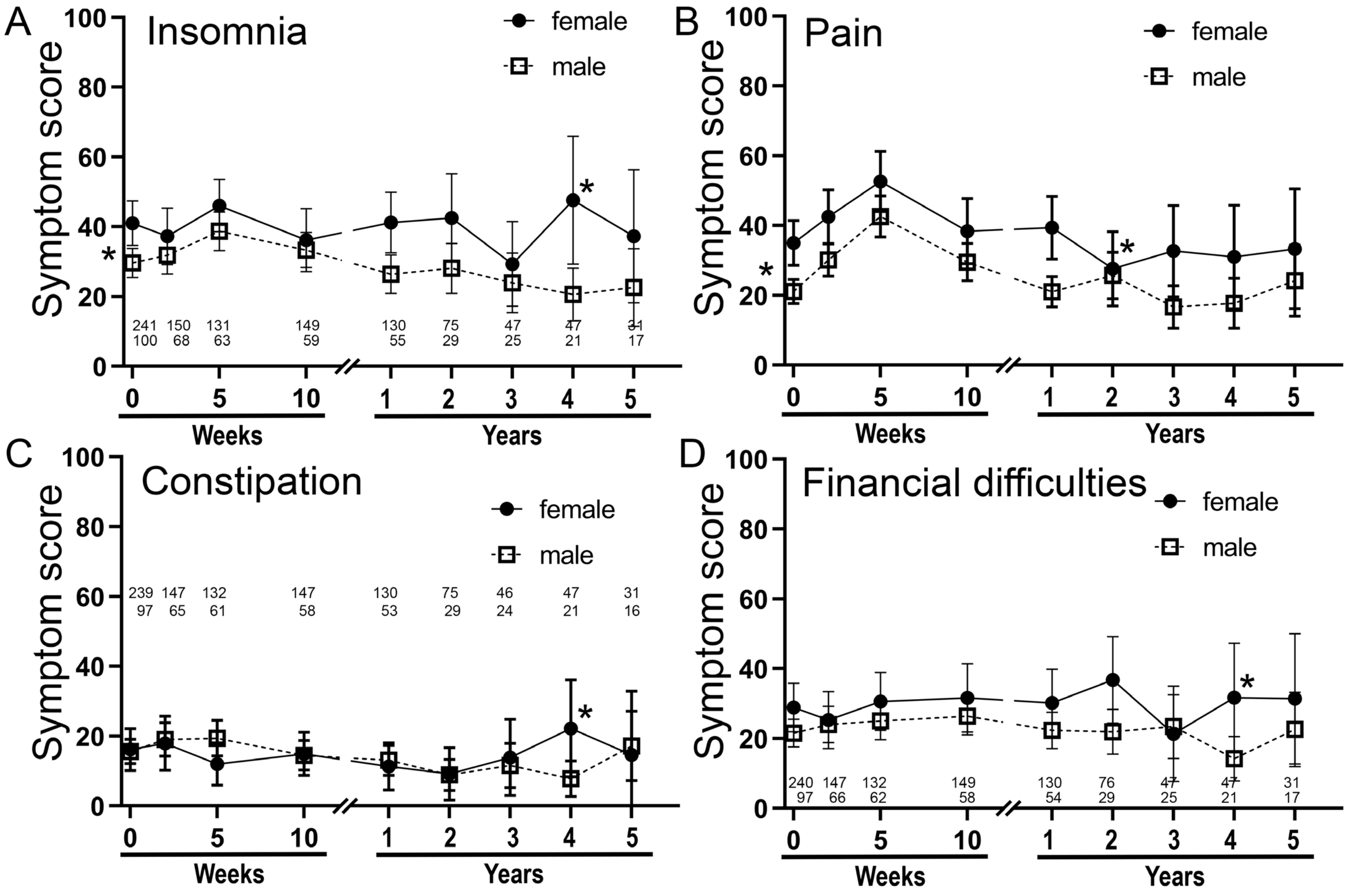

Supplementary figure 8

Supplementary Figure S8: Symptom scores of quality of life by QLQ C30 questionnaires (Quality of live cohort). Data points are baseline, during RCT (2nd and 3rd point), directly prior to surgery (4th point) and 1 to 5 years after beginning of RCT. (A) Insomnia (B) pain (C) constipation and (D) financial difficulties. Asterisks to the left of the abscissa mark differences of more than 10% in the baseline and asterisks in the time data mark a change of more than 10 percentage points from the baseline. The length of the error bars corresponds to the 95% confidence interval for the mean. The number of patients who answered the questionnaires is given.

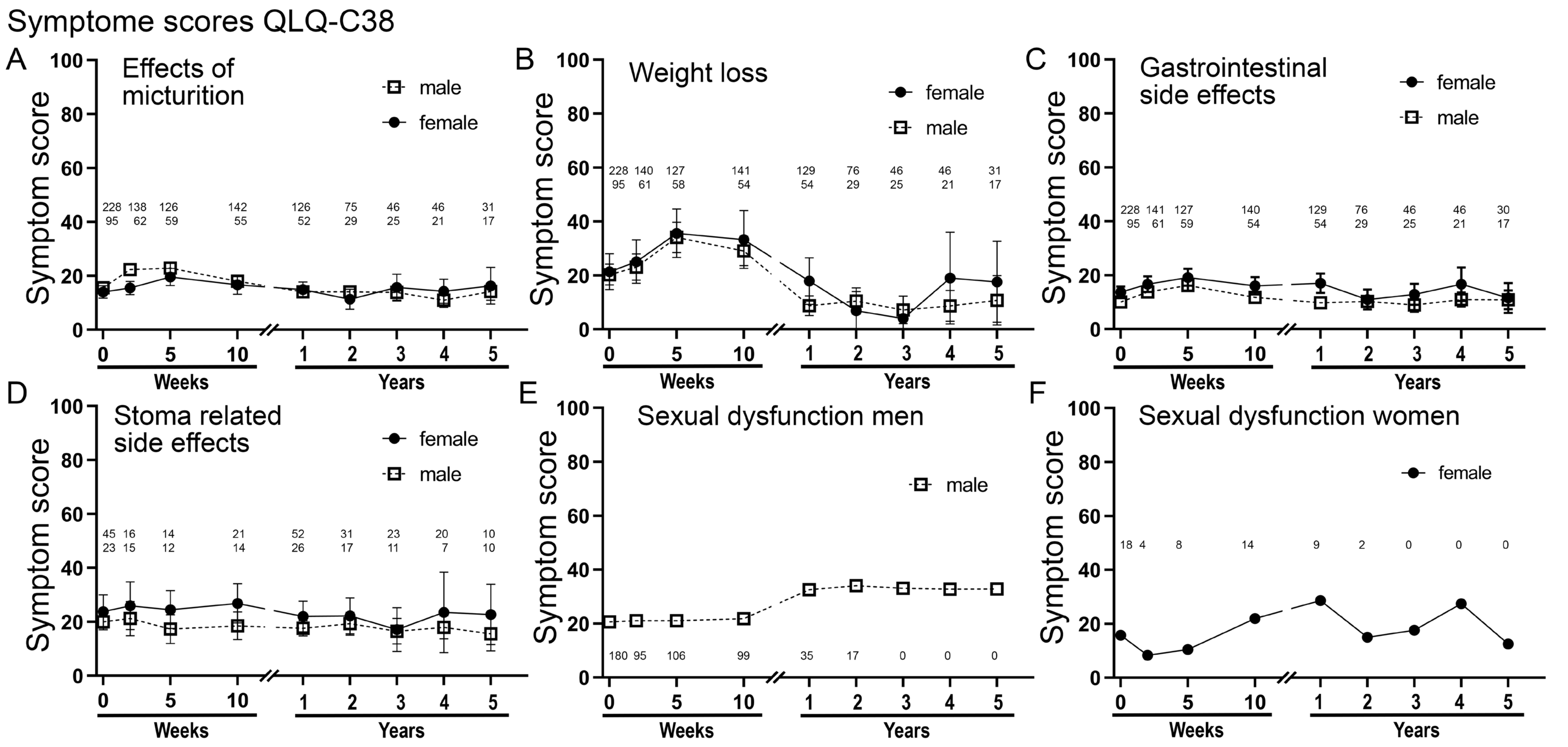

Supplementary Figure S9: Symptom scores of quality of life by QLQ CR38 questionnaires (Quality of live cohort). Data points are baseline, during RCT (2nd and 3rd point), directly prior to surgery (4th point) and 1 to 5 years after beginning of RCT. (A) Effects of micturition, (B) weight loss, (C) gastrointestinal side effects, (D) stoma related side effects, (E) sexual dysfunction men and (F) sexual dysfunction women. Asterisks to the left of the abscissa mark differences of more than 10% in the baseline and asterisks in the time data mark a change of more than 10 percentage points from the baseline. The length of the error bars corresponds to the 95% confidence interval for the mean. The number of patients who answered the questionnaires is given.
